# Clinical and Radiological Outcomes of Acute Type A Aortic Dissection Repair with the Ascyrus Medical Dissection Stent

**DOI:** 10.3390/jcm14238553

**Published:** 2025-12-02

**Authors:** Francesco Cabrucci, Beatrice Bacchi, Dario Petrone, Massimo Baudo, Dimitrios E. Magouliotis, Yoshiyuki Yamashita, Serge Sicouri, Massimo Bonacchi, Sandro Gelsomino, Basel Ramlawi

**Affiliations:** 1 Department of Cardiac Surgery Research, Lankenau Institute for Medical Research, Main Line Health, Wynnewood, PA 19096, USA; massimo.baudo@icloud.com (M.B.); magouliotisd@mlhs.org (D.E.M.); yoshiyuki.yamashita.md@gmail.com (Y.Y.); sicouris@mlhs.org (S.S.); ramlawib@mlhs.org (B.R.); 2 Cardiothoracic Department, CARIM School for Cardiovascular Diseases, Maastricht University, 6211 LH Maastricht, The Netherlands; beatricebacc@gmail.com (B.B.); sandro.gelsomino@maastrichtuniversity.nl (S.G.); 3 Cardiac Surgery Unit, Department of Experimental and Clinical Medicine, University of Florence, 50121 Firenze, Italy; dario.petrone@unifi.it (D.P.); massimo.bonacchi@unifi.it (M.B.); 4 Department of Cardiac Surgery, Lankenau Medical Center, Main Line Health, Wynnewood, PA 19096, USA

**Keywords:** type A aortic dissection, hybrid arch repair, Ascyrus Medical Dissection Stent, hypothermic circulatory arrest, DANEs, false lumen remodeling

## Abstract

**Objective:** This study aimed to evaluate clinical and radiological outcomes of Ascyrus Medical Dissection Stent (AMDS^®^, Artivion Inc.) for acute type A aortic dissection (ATAAD). **Methods:** Between January 2021 and January 2025, all consecutive patients undergoing emergent surgery for ATAAD and hybrid aortic arch repair using the AMDS from two centers were retrospectively analyzed. Demographic, intraoperative, and postoperative data were collected. Patients were stratified based on 30-day or in-hospital mortality. Survival analysis was performed for patients who survived hospital discharge. Radiological evaluation focused on the presence of distal anastomotic new entries (DANEs), false lumen thrombosis, and aortic remodeling on follow-up computed tomography angiography. A total of 46 patients (12 female, mean age 66.1 ± 13.8 years) were included in the study. **Results:** The 30-day or in-hospital mortality rate was 21.7% (10 patients). There were no significant differences in demographic variables between survivors and non-survivors. All patients underwent hemiarch replacement with AMDS stent placement, with 54.3% also requiring aortic root replacement. Median cross-clamp time, hypothermic circulatory arrest (HCA) time, and time of antegrade selective cerebral perfusion did not differ significantly between the two groups. However, significant differences were observed in median cardiopulmonary bypass (CPB) time (151 vs. 274 min, *p* = 0.02) and HCA temperature (27 °C vs. 25 °C, *p* = 0.021). Postoperatively, the non-survivor group showed a significantly higher incidence of dialysis requirement (7.7% vs. 60.0%, *p* = 0.02), use of mechanical circulatory support (3.9% vs. 44.4%, *p* = 0.01), and re-exploration for bleeding (15.4% vs. 66.7%, *p* = 0.023). **Conclusions:** The AMDS^®^ is an effective adjunct in hemiarch replacement for ATAAD. Moderate hypothermia and optimized perfusion were linked to better early survival, while the device reliably promoted true-lumen expansion with few DANEs. Its rapid deployment may further facilitate the use of moderate hypothermia by balancing procedural efficiency with systemic protection.

## 1. Introduction

Acute type A aortic dissection (ATAAD) carries a time-dependent mortality risk, with an estimated increase of 0.5% per hour if left untreated. Surgical intervention has demonstrated unequivocal superiority over medical management [1]. Over the past two decades, thanks to significant advancements in diagnostic modalities, operative techniques, organ-protection strategies, and hybrid approaches, the historically high postoperative mortality has declined substantially, now ranging between 15% and 18% [2]. The refinement of the hybrid technique, coupled with continuous advancements in device technology driven by industry, holds promise to reduce mortality even further [3,4]. The Ascyrus Medical Dissection Stent (AMDS; Artivion^®^, Atlanta, GA, USA), one of the latest devices released, has shown a significant potential to transform the management of ATAAD [5]. The AMDS, an uncovered nitinol wire braided stent anchored to a PTFE felt sewing cuff, was created to exert a radial force inside the true lumen (TL) to stabilize it and reduce the dynamic malperfusion [6]. Furthermore, the device promotes positive aortic remodeling, which is essential for long-term durability and prevention of aneurysmal degeneration. The AMDS is also characterized by its ease of use, requiring minimal additional operative time and integrating seamlessly into standard hemiarch procedures, thereby making it an attractive adjunct in selected ATAAD cases.

Our research aims to (1) evaluate the early clinical and radiological outcomes of hybrid aortic arch repair using the AMDS^®^ in patients with ATAAD, (2) identify perioperative predictors of 30-day or in-hospital mortality, and (3) explore factors associated with favorable false lumen remodeling during follow-up. 

## 2. Methods

Between January 2021 and January 2025, all consecutive patients referred for acute type A aortic dissection (ATAAD) were retrospectively reviewed at two institutions—one located in Europe and the other in the United States of America.

Inclusion criteria were (1) patients undergoing open aortic surgery with ascending aorta and/or hemiarch replacement using hypothermic circulatory arrest (HCA), (2) those who received hybrid aortic arch repair with the Ascyrus Medical Dissection Stent (AMDS^®^, Artivion Inc.), (3) irrespective of concomitant cardiac procedures, (4) including all tiers of surgical priority, and (5) those who had undergone previous cardiac surgery.

Exclusion criteria included (1) patients who underwent ascending aorta and/or hemiarch replacement without using the AMDS prosthesis, (2) those who received total arch replacement using conventional, elephant trunk, or frozen elephant trunk techniques, and (3) those who refused surgical treatment.

After applying the selection criteria, 46 out of 204 patients were included in the current analysis. Demographic, intraoperative, and postoperative data were retrospectively collected and analyzed. Patients were stratified based on 30-day or in-hospital mortality. Conditional survival analysis was performed for patients who survived beyond hospital discharge.

### 2.1. AMDS: Technique, Indications, and Contraindications

An extensive technical overview of a step-by-step implantation of the AMDS device has previously been described [7,8]. This section aims to emphasize several core concepts that have emerged as particularly relevant based on our experience, some of which will be further explored in the Discussion section.

Given its design characteristics, the AMDS is best indicated in ATAAD with the entry tear located in the aortic root or ascending aorta, particularly when there is radiological or clinical evidence of dynamic malperfusion. Although the indications may appear broad, surgeons should refrain from employing this stent in all comers, as it is not intended to function as a ‘one-size-fits-all’ device [9]. Critical contraindications must be considered, such as tears involving the aortic arch or supra-aortic branches, true aneurysmal dilatation of the arch or descending aorta, and the presence or strong suspicion of connective tissue disorders [7,9]. Based on our experience, the mere suspicion of static malperfusion affecting the supra-aortic vessels represents a relative contraindication, typically prompting the consideration of more comprehensive approaches such as total arch replacement or debranching procedures. Finally, drawing from our practice and learning curve, the most critical recommendation is to adhere strictly to the proposed implantation technique (leaving at least 10 mm of aortic tissue before the innominate artery) and to avoid beveling the aortic transection toward the inner curvature of the arch.

### 2.2. Radiological Follow-Up

At least one computed tomography angiography (CTA) scan was available for all patients who survived to hospital discharge. The timing of postoperative CTA assessments was not standardized across centers; therefore, when multiple scans were available, different scans were selected for specific analyses. Distal anastomosis new entries (DANEs) were evaluated using the earliest postoperative CTA, in accordance with established literature on this topic [10]. False lumen (FL) remodeling, instead, was assessed using the CTA with the longest available follow-up for each patient, with a required minimum interval of 3 months after surgery to ensure meaningful evaluation of early aortic remodeling. FL status was categorized into three groups—FL perfused, FL partially perfused, and FL thrombosed—based on comparative enhancement between FL and true lumen (TL) in the medial portion of the descending aorta. An FL was considered perfused if its opacification was similar to that of the TL. If the FL showed less than 50% of the Hounsfield units of the TL, it was defined as partially perfused. In cases where the FL exhibited no contrast enhancement, it was classified as thrombosed.

### 2.3. Statistical Analysis

Continuous variables are summarized as mean ± standard deviation or median (interquartile range), contingent upon normality as determined by the Shapiro–Wilk test. Categorical variables are presented as frequencies (percentages). Differences in normally distributed variables were assessed using the *t*-test, while non-normally distributed variables were analyzed with the Mann–Whitney *U* test. Categorical variables were compared using the Chi-square test or Fisher’s test, as needed.

Associations between clinical variables and 30-day mortality were assessed using univariate logistic regression with Firth’s penalized likelihood to account for the small sample size and prevent bias due to separation. Given the limited number of events (n = 10), multivariable analysis was not performed to avoid model overfitting. Survival analysis was conducted using the Kaplan–Meier survival function.

Statistical analyses were conducted in R and RStudio (Version: 2024.12.0+467) employing “TableOne”, “Logistif”, “Survimer”, and “ggplot2” packages.

## 3. Results

### 3.1. Early Clinical Outcomes

A total of 46 patients were included in the analysis, comprising 15 females (32.6%), with a mean age of 67 ± 13.6 years. Preoperatively, 31 patients (67.4%) had a history of hypertension, and 3 (6.5%) had chronic kidney disease (CKD). Upon arrival at the hub hospital, 4 patients (8.7%) were intubated, and 2 (4.3%) presented in acute shock. Pericardial effusion was observed in 9 patients (19.6%), while clinical signs of end-organ or limb malperfusion were present in 24 patients (52.2%). The most frequent location of the intimal tear was the ascending aorta (74%), followed by the aortic root (26%). In the majority of patients (43%), the false lumen extended from the ascending aorta (Zone 0) distally beyond Zone 9, involving the iliac or lower limb arteries. All preoperative and demographic variables are summarized in Table 1.

Axillary artery arterial and bicaval venous cannulation were the most utilized cardiopulmonary bypass (CPB) configurations, employed in 84.8% and 63% of cases, respectively. All patients underwent ascending aorta replacement under HCA with successful AMDS deployment. The most used AMDS size was the tapered 55–40 mm configuration, implanted in 20 patients (43.5%). Aortic root replacement was performed in 20 patients (43.5%), while additional concomitant procedures were carried out in 5 patients (10.9%). A complete list of all intraoperative variables is shown in Table 2.

In our study, the cumulative 30-day and in-hospital mortality was 21.7%, with 7 patients (15.2%) dying within 30 days postoperatively and an additional 3 patients (6.5%) dying in-hospital before discharge. Among the 7 patients who died within 30 days, 2 succumbed to multiorgan failure, 2 to brain death following a cerebrovascular accident, 1 to mesenteric ischemia, 1 to uncontrollable bleeding secondary to coagulopathy, and 1 to heart failure due to right coronary artery malperfusion occurring in the preoperative period. Mechanical circulatory support (MCS) was required in 5 patients (10.9%): 4 received veno-arterial extracorporeal membrane oxygenation (VA-ECMO), and 1 was supported with a temporary right ventricular assist device (RVAD) using a ProtekDuo^®^ cannula (LivaNova plc, London, UK). Of the patients on VA-ECMO, only one was successfully weaned and survived to hospital discharge. Detailed postoperative outcomes are summarized in Table 3.

Stratification analysis between survivors and non-survivors revealed no significant differences in demographic or preoperative variables, except for preoperative intubation, which was more frequent among non-survivors (3 patients, 30%) compared to survivors (1 patient, 2.8%; *p* = 0.039). No differences were observed between the two groups in procedural technical aspects. However, significant differences were found in intraprocedural parameters: serum lactate levels were significantly lower in survivors compared to non-survivors, both pre-CPB (1.83 ± 1.13 vs. 5.08 ± 5.32 mmol/L, *p* = 0.002) and post-CPB (4.50 ± 1.42 vs. 10.86 ± 4.83 mmol/L, *p* < 0.001). Similarly, significant differences appeared in the post-CPB level of hemoglobin (9.08 ± 1.48 vs. 6.88 ± 0.68, *p* < 0.001) and hematocrit (27.99 ± 4.60 vs. 20.91 ± 2.01, *p* < 0.001) between the two groups.

Interestingly, a statistically significant difference was observed in the temperature during HCA, with survivors averaging 28.04 ± 2.32 °C compared to 24.20 ± 2.62 °C in non-survivors (*p* < 0.001). Additionally, autologous blood reinfusion from the cell saver was administered to only 1 patient (2.8%) in the survivor group, whereas 6 patients (60%) in the non-survivor group received cell-salvaged blood (*p* < 0.001). All differences between the survivor and non-survivor groups are detailed in Table 1, Table 2 and Table 3.

Univariate Firth’s logistic regression analysis identified several predictors significantly associated with 30-day or in-hospital mortality (Table 4). Notably, higher HCA temperature was associated with increased survival (OR 0.45, 95% CI 0.22–0.72; *p* < 0.001), whereas the use of autologous blood was strongly associated with mortality (OR 34.2, 95% CI 5.50–91.81; *p* < 0.001).

### 3.2. Follow-Up and Radiological Outcomes

**Figure 1:**
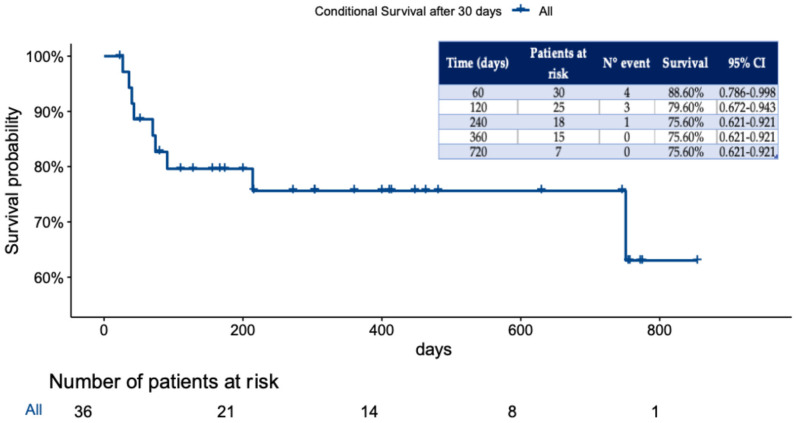
Kaplan–Meier curve showing the conditional survival function for the study population. Only patients who survived after 30 days or were discharged from the hospital are shown.

In our study, the overall conditional survival beyond 30-day or in-hospital mortality was 75.6%, with a median follow-up of 244 days (IQR, 88–467), as shown in Figure 1.

DANEs were identified in only 4 patients (11.1%), and AMDS midportion coiling was reported in 2 patients (5.6%). Longer evaluation of CTA revealed complete thrombosis in 21 patients (58.3%), while partial thrombosis was seen in 9 (25.0%) patients and persistent FL perfusion in 6 (16.7%) patients (Table 5).

## 4. Discussion

The present study evaluated early clinical and radiological outcomes after hybrid aortic arch repair using the AMDS^®^ in patients with ATAAD. Our findings confirm that AMDS implantation during standard hemiarch replacement is safe and feasible, providing true lumen stabilization and promoting favorable false lumen remodeling, with perioperative mortality rates comparable to those reported in contemporary series.

### 4.1. Predictors of Early Mortality

The 30-day or in-hospital mortality rate of 15.2% in our cohort aligns with previous multicenter experiences, including the DARTS trial, which reported a 13.7% 30-day mortality in 342 patients across nine centers [11]. Although our study included a smaller, bicentric cohort compared with the large multicenter DARTS registry, the comparable rates of mortality and false lumen thrombosis (15.2% vs. 13.7% and 83.3% vs. 84%, respectively) suggest that the benefits associated with AMDS implantation are reproducible across different institutional settings. Like DARTS, our data support the device’s capacity to promote early aortic remodeling and to reduce distal malperfusion. However, we further identified intraoperative physiologic predictors—particularly HCA, CPB duration, and lactate levels—as determinants of early mortality, thereby adding a hemodynamic dimension to the structural findings emphasized by the registry.

Lower HCA temperatures (<25 °C) were associated with worse outcomes. Although deep hypothermia has been widely adopted for cerebral protection, excessive cooling prolongs CPB and reperfusion times, aggravates coagulopathy, and may heighten systemic inflammatory response. Conversely, moderate hypothermia (26–28 °C) combined with selective antegrade cerebral perfusion ensures effective cerebral protection while facilitating faster recovery and improved hemostasis. These observations are consistent with recent evidence suggesting that colder is not necessarily safer in complex arch surgery [2,3].

In this context, the rationale for favoring moderate hypothermia during AMDS-assisted repair is further strengthened by technical aspects of the procedure: AMDS deployment adds minimal time to the construction of the distal anastomosis, thereby reducing circulatory arrest and overall CPB duration compared with more extensive arch repairs. This procedural efficiency may lessen the need for deep hypothermia and support a more physiological temperature-management strategy. Given the limited sample size and retrospective design of our study, however, this association should be interpreted cautiously. The observed relationship between higher HCA temperatures and improved early outcomes may reflect a trend rather than a definitive effect, and unmeasured confounding factors are likely to contribute. As such, no causal inference can be drawn, and confirmation in larger prospective cohorts will be essential.

Additionally, elevated pre- and post-CPB lactate levels and longer CPB times predicted mortality, highlighting the critical importance of perfusion optimization. Some predictors—particularly autologous blood reinfusion—showed very large odds ratios accompanied by wide confidence intervals, reflecting the limited sample size and the inherent instability of estimates derived from rare events. These findings should therefore be interpreted with caution. The apparent association between autologous blood reinfusion and early mortality is unlikely to represent a true causal relationship; rather, it more plausibly reflects its use as a rescue strategy in cases of severe intraoperative bleeding.

### 4.2. Radiological and Midterm Outcomes

**Figure 2:**
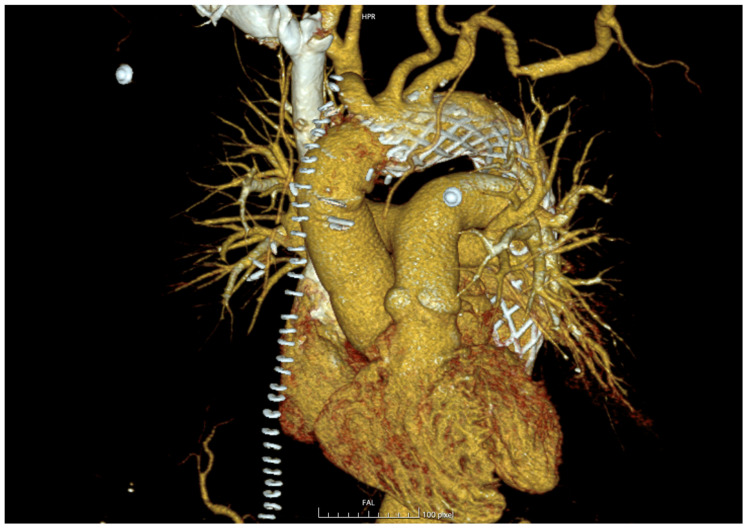
Three-dimensional postoperative CT reconstruction of the thoracic aorta showing an AMDS implanted at the aortic arch.

Follow-up imaging (Figure 2) demonstrated encouraging aortic remodeling, with complete or partial false lumen thrombosis in 83.3% of survivors and a low rate of distal anastomosis new entries (DANEs, 11.1%), consistent with the DARTS trial and other recent series [5,7,10]. These findings confirm that meticulous adherence to the recommended implantation technique—specifically maintaining at least 10 mm of native aortic tissue proximal to the innominate artery and avoiding beveling—minimizes distal complications.

Recent data [11] have further suggested that AMDS implantation significantly reduces postoperative DANE formation. Nevertheless, as highlighted in the Canadian study [11], the durability of these benefits over time remains to be confirmed, and long-term surveillance is essential to determine whether improved distal sealing truly decreases the need for late reinterventions.

### 4.3. Clinical Implications

The AMDS offers a valuable adjunct for ATAAD cases with a primary entry tear in the ascending aorta and dynamic malperfusion. Its integration into standard hemiarch repair allows stabilization of the distal true lumen without the increased morbidity of total arch replacement. Nevertheless, appropriate patient selection remains crucial. The presence of connective-tissue disorders, arch tears, or true aneurysmal degeneration continues to warrant more extensive approaches such as frozen-elephant-trunk or total-arch replacement.

Hybrid and endovascular strategies for aortic arch repair increasingly recognize the AMDS^®^ as an effective bridge between conventional hemiarch replacement and total arch replacement [4]. Reported outcomes demonstrate comparable survival and favorable aortic remodeling, with results strongly influenced by cerebral protection strategy and institutional experience. In line with these observations, our findings further suggest that refinements in intraoperative management—particularly the adoption of moderate hypothermia and optimized perfusion parameters—may substantially contribute to improved early outcomes following AMDS-assisted hybrid arch repair.

**Figure 3:**
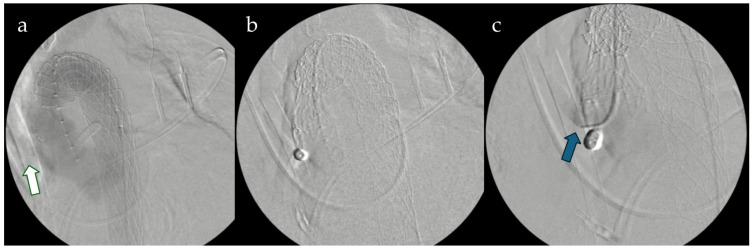
Intraoperative fluoroscopic sequences showing (**a**) angiographic result of AMDS and de-branching of the supra-aortic vessels via a trifurcated graft 12 × 8 × 8 mm connected to the ascending aorta (white arrow). (**b**) 37 mm Gore TAG endograph is expanding inside the AMDS. (**c**) Close-up vision during TEVAR deployment with the angiographic catheter inside the trifurcated graft (blue arrow).

Furthermore, the AMDS architecture inherently lends itself to potential secondary interventions, including thoracic endovascular aortic repair (TEVAR) within the previously implanted stent. This approach has been described in the literature as both a planned second-stage procedure and a rescue option in cases of persistent distal false lumen perfusion [5]. Although only 2.1% of patients in our cohort required subsequent endovascular treatment, these observations reinforce the concept of a *step-up hybrid strategy* when distal remodeling remains incomplete and highlight the mechanical compatibility between the uncovered AMDS scaffold and downstream covered stent grafts (Figure 3).

Collectively, these studies converge on three fundamental principles:

(1) Careful patient selection and technical precision are essential to minimize distal complications;
(2) Hybrid repair with AMDS effectively promotes true-lumen expansion and favorable remodeling while avoiding the morbidity of total arch replacement;
(3) Sequential endovascular completion can safely address residual false-lumen perfusion when necessary (Figure 3).

By incorporating systemic physiologic determinants of early mortality into the assessment of AMDS performance, our study extends current evidence by linking procedural technique with perfusion physiology as complementary drivers of early postoperative outcomes. Moreover, the simplicity and rapidity of AMDS deployment—which requires no additional complex operative steps—may further facilitate the routine use of moderate hypothermia strategies, thereby enhancing systemic protection without compromising procedural efficiency.

## 5. Limitations

The retrospective, bicentric design and limited sample size constrain the generalizability of our conclusions, and the imaging follow-up schedule was not standardized. A major limitation of our study is the absence of a comparative cohort treated with standard hemiarch repair without the AMDS^®^ (or with an alternative stent), which restricts our ability to draw causal inferences regarding the device’s incremental benefit. Due to the small sample size and low event rate, Firth’s logistic regression was applied to reduce small-sample bias. This approach occasionally yielded wide confidence intervals for certain predictors, particularly for variables such as autologous blood reinfusion—reflecting limited statistical precision rather than true effect magnitude. These results should therefore be interpreted with caution, as the observed associations may represent markers of underlying clinical instability rather than genuine causal relationships. Prospective multicenter studies are warranted to validate these predictors of early mortality, refine optimal temperature management, and assess the long-term durability of AMDS-mediated aortic remodeling.

## 6. Conclusions

The AMDS^®^ represents a safe and effective adjunct during hemiarch replacement for ATAAD. In our experience, moderate hypothermia and optimized perfusion management were associated with improved early survival. The device promoted consistent true-lumen expansion and a low incidence of distal anastomotic new entries. These findings align with multicenter data suggesting that AMDS contributes to early aortic remodeling and reduced distal re-entry formation, although its long-term impact on the need for secondary aortic interventions remains to be determined. Although long-term data are still required, these findings support the growing role of the AMDS^®^ as a key component of modern hybrid management for acute aortic dissection.

## Figures and Tables

**Table 1:** Preoperative variables.

	30-Day or In-Hospital Mortality			
	Survived	Deceased		Total
	n = 36	n = 10	*p*	n = 46
**Age** (Mean/SD)	65.81 (13.12)	71.60 (15.06)	0.238	67.07 (13.61)
**Female**	10 (27.8)	5 (50.0)	0.345	15 (32.6)
**Comorbidities**				
Hypertension	25 (69.4)	6 (60.0)	0.855	31 (67.4%)
Dyslipidemia	8 (22.2)	1 (10.0)	0.681	9 (19.6%)
Active Smoking	11 (30.6)	2 (20.0)	0.796	13 (28.3%)
Diabetes	2 (5.6)	1 (10.0)	1	3 (6.5%)
AF	4 (11.1)	2 (20.0)	0.835	6 (13.0%)
COPD	3 (8.3)	1 (10.0)	1	4 (8.7%)
CKD	2 (5.6)	1 (10.0)	1	3 (6.5%)
**Previous Cardiac Surgery**	0 (0.0)	2 (20.0)	0.062	2 (4.3%)
**Clinical Presentation**				
Intubation	1 (2.8)	3 (30.0)	**0.039**	4 (8.7%)
Acute Shock	1 (2.7)	1 (10.0)	0.462	2 (4.3%)
Acute Neurological Deficit	3 (8.3)	0 (0.0)	0.826	3 (6.5%)
Clinical Malperfusion	17 (47.2)	7 (70.0)	0.202	24 (52.2%)
LVEF % (mean/SD)	51.23 (6.99)	51.00 (11.40)	0.954	51.79 (7.71)
Severe Aortic Regurgitation	16 (46)	5 (50)	0.729	21 (45.6%)
Pericardial Effusion	7 (19.4)	2 (20.0)	1	9 (19.6%)
**Extension of Dissection**	-	-	0.7	
Ascending, arch, descending, including limb (beyond Zone 9)	16 (44.4)	3 (30.0)		19 (41.3%)
Ascending, arch, descending (up to Zone 9)	15 (41.7)	5 (50.0)		20 (43.5%)
Ascending, arch, high descending (up to Zone 3)	5 (13.9)	2 (20.0)		7 (15.2%)
**Location of the Tear**	-	-	0.403	
Root	10 (27.8)	2 (20)		12 (26%)
Ascending aorta	26 (72.2)	8 (90)		34 (74%)
Arch	0 (0.0)	0 (0.0)		0 (0)

AF—atrial fibrillation; COPD—chronic obstructive pulmonary disease; CKD—chronic kidney disease; LVEF—left ventricular ejection fraction; SD—standard deviation; the bold *p* value indicates statistical significance (*p* < 0.05).

**Table 2:** Intraoperative variables.

	30-Day Mortality or In-Hospital			
	Survived	Deceased		Total
	n = 36	n = 10	*p*	n = 46
**Arterial Cannulation (n%)**			0.195	
Axillary	31 (86.1)	8 (80.0)		39 (84.8%)
Femoral	2 (5.6)	1 (10.0)		3 (6.5%)
Arch	3 (8.3)	1 (0.0)		4 (8.6%)
**Venous Cannulation (n%)**			0.199	
Bicaval	25 (69.4)	4 (40.0)		29 (63.0%)
Femoral	6 (16.7)	4 (40.0)		10 (21.7%)
Right Atrium	5 (13.9)	2 (20.0)		7 (15.2%)
**Ascending Aorta Replacement/hemiarch**	36 (100%)	10 (100%)		46 (100%)
**AMDS (n%)**			0.597	
40–30	3 (8.3)	1 (10.0)		4 (8.7%)
40–40	10 (27.8)	2 (20.0)		12 (26.1%)
40–55	1 (2.8)	0 (0.0)		1 (2.2%)
55–40	16 (44.4)	4 (40.0)		20 (43.5%)
55–50	1 (2.7)	1 (10.0)		2 (4.3%)
55–55	5 (13.9)	2 (20.0)		7 (15.2%)
**Aortic Valve Procedures (n%)**			0.582	
Not performed	9 (25.0)	1 (10.0)		10 (21.7%)
Replacement	19 (52.8)	6 (60.0)		25 (54.3%)
Repair/Resuspension	8 (22.2)	3 (30.0)		11 (23.9%)
**Root Operation (n%)**			0.173	
Not performed	14 (38.9)	1 (10.0)		15 (32.6%)
Bentall	15 (41.7)	5 (50.0)		20 (43.5%)
Bavaria	7 (19.4)	4 (40.0)		11 (23.9%)
**Concomitant Surgery**	2 (5.6)	3 (30.0)	0.105	5 (10.9%)
**Autologous Blood** (Cell saver)	1 (2.8)	6 (60.0)	**<0.001**	7 (15.2%)
Hb Pre CPB mg/dL, (mean/SD)	11.53 (2.17)	10.73 (1.85)	0.294	11.36 (2.11)
Hb Post CPB mg/dL, (mean/SD)	9.08 (1.48)	6.88 (0.68)	**<0.001**	8.65 (1.62)
Lactate Pre CPB, mmol/L (mean/SD)	1.83 (1.13)	5.08 (5.32)	**0.002**	2.46 (2.77)
Lactate Post CPB, mmol/L (mean/SD)	4.50 (1.42)	10.86 (4.83)	**<0.001**	5.74 (3.50)
HCT Pre CPB % (mean/SD)	34.24 (5.98)	31.59 (5.38)	0.26	33.72 (5.90)
HCT Post CPB % (mean/SD)	27.99 (4.60)	20.91 (2.01)	**<0.001**	26.61 (5.07)
**CPB Time**, minutes, (median/IQR)	143.00 [130.00, 166.25]	265.00 [162.25, 345.75]	**0.004**	150.00 [131.50, 191.25]
**Cross Clamp Time**, minutes, (mean/SD)	104.61 (26.73)	129.80 (71.18)	0.118	112.26 (42.24)
**HCA Time**, minutes (mean/SD)	31.44 (8.80)	29.60 (6.98)	0.545	31.04 (8.40)
**Selective Antegrade Cerebral Perfusion Time**, minutes (mean/SD)	22.83 (10.48)	22.60 (8.88)	0.949	22.78 (10.06)
**Reperfusion Time**, minutes, (median [IQR])	24.00 [21.00, 32.00]	50.50 [23.00, 102.75]	0.087	26.00 [22.00, 34.00]
**HCA Temperature** °C, (mean/SD)	28.04 (2.32)	24.20 (2.62)	**<0.001**	27.13 (2.89)

AMDS—Ascyrus Medical Dissection Stent; Hb—hemoglobin; CPB—cardiopulmonary bypass; HCT—hematocrit; HCA—hypothermic circulatory arrest; SD—standard deviation; IQR—interquartile range; the bold *p* value indicates statistical significance (*p* < 0.01).

**Table 3:** Postoperative variables.

	30-Day Mortality or In-Hospital			
	Survived	Deceased		Total
	n = 36	n = 10	*p*	n = 46
**ICU LOS** days (median/IQR)	10.00 [4.00, 32.25]	3.00 [2.00, 4.50]	-	8.00 [3.00, 24.00]
**Intubation time** hours (median/IQR)	72.00 [14.50, 162.00]	61.75 [24.98, 108.88]	-	72.00 [15.50, 144.00]
**Respiratory Failure**	16 (44.4)	2 (33.3)	0.949	18 (39.1%)
**Tracheostomy**	11 (30.6)	0 (0.0)	-	11 (23.9%)
**CRRT**	5 (14.7)	4 (66.7)	**0.023**	5 (16.1%)
**Intestinal Ischemia**	2 (5.9)	2 (33.3)	0.184	4 (8.7%)
**New Stroke**	2 (5.5%)	1 (10.0%)	0.422	3 (6.5%)
**Re-exploration for bleeding**	4 (15.4%)	4 (66.7%)	**0.023**	8 (17.3%)
**Endovascular repair** (post AMDS deployment)	0 (0%)	1 (10%)	0.186	1 (2.1%)
**MCS**	1 (3.9%)	4 (44.4%)	**0.01**	5 (10.8%)

*p*-value for ICU LOS, intubation time, and tracheostomy was not calculated due to the inherent differences in survivors vs. non-survivors. ICU LOS—intensive care unit length of stay; CRRT—continuous renal replacement therapy; MCS—mechanical circulatory support; IQR—interquartile range; the bold *p* value indicates statistical significance (*p* < 0.05).

**Table 4:** Univariate Firth’s logistic regression analysis of predictors of 30-day mortality or in-hospital mortality.

Variable	OR	95% CI	*p*-Value
**HCA Temperature** (each °C)	0.45	0.22–0.72	<0.001
**Autologous Blood Use**	34.2	5.50–91.81	<0.001
**CPB Time** (per minute)	1.02	1.01–1.04	<0.001
Lactate Level Pre-CPB	1.36	1.07–2.44	<0.001
Lactate Level Post-CPB	1.92	1.34–3.69	<0.001

CPB—cardiopulmonary bypass; HCA—hypothermic circulatory arrest; OR—odds ratio; CI—confidence interval.

**Table 5:** Clinical and radiological follow-up parameters.

Clinical and Radiological Follow-Up	
**Follow-up time**, days (median [IQR])	244 [88.2, 467.5]
**Follow-up mortality** (%)	9 (25.0)
False lumen **perfused** (%)	6 (16.7)
False lumen **partially perfused** (%)	9 (25.0)
False lumen **thrombosed** (%)	21 (58.3)
**DANEs** (%)	4 (11.1)
AMDS **Midportion coiling** (%)	2 (5.6)

Only patients who survived after 30 days or were discharged from the hospital are shown. DANEs—distal anastomosis new entry tears; AMDS—Ascyrus Medical Dissection Stent.

## Data Availability

The raw data supporting the conclusions of this article will be made available by the authors upon request and institutional approval.

## Abbreviations

AMDS	Ascyrus Medical Dissection Stent
ATAAD	Acute type A aortic dissection
DANEs	Distal anastomotic new entries
DARTS	Dissected aorta repair through stent implantation
FET	Frozen elephant trunk
TEVAR	Thoracic endovascular aortic repair
